# Wirksamkeit einer Intervention zur Verringerung der Stigmatisierung von Menschen mit Hauterkrankungen unter Fachkräften im Gesundheits‐ und Körperpflegebereich – eine randomisierte kontrollierte Studie

**DOI:** 10.1111/ddg.16017_g

**Published:** 2026-08-04

**Authors:** Juliane Traxler, Catharina C. Braren‐von Stülpnagel, Matthias Augustin, Marius Grosser, Dennis Roggenkamp, Rachel Sommer

**Affiliations:** ^1^ Institut für Versorgungsforschung in der Dermatologie und bei Pflegeberufen (IVDP) Universitätsklinikum Hamburg‐Eppendorf (UKE), Hamburg; ^2^ Deutscher Psoriasis Bund e.V., Hamburg; ^3^ Beiersdorf AG, Hamburg

**Keywords:** Hautkrankheiten, Intervention, Psoriasis, RCT, Stigmatisierung, Intervention, psoriasis, RCT, skin diseases, stigmatization

## Abstract

**Hintergrund und Zielsetzung:**

Menschen mit Hauterkrankungen werden in der Gesundheitsversorgung und Körperpflege häufig stigmatisiert, was ihre Lebensqualität erheblich beeinträchtigt. Diese randomisierte kontrollierte Parallelgruppenstudie evaluierte ein Präsenzseminar, das darauf abzielte, diese Stigmatisierung unter Fachkräften des Gesundheits‐ und Körperpflegebereichs (FGKP) zu verringern.

**Patienten und Methodik:**

Kosmetikerinnen, Friseure, Pflegekräfte und Physiotherapeutinnen wurden randomisiert einer Interventionsgruppe (IG; n = 64) oder einer Kontrollgruppe (KG; n = 65) zugeordnet. Die IG nahm an einem Seminar teil, das Selbstreflexionsübungen, Aufklärung und eine Begegnung mit einem Patienten umfasste; die KG erhielt ein Seminar zum Thema „Gesundheit am Arbeitsplatz”. Zustimmung zu negativen Stereotypen, krankheitsbezogene Fehlannahmen, Wunsch nach sozialer Distanz und Verhaltensabsichten wurden zu Beginn (t0), nach der Intervention (t1) und nach 3 Monaten (t2) bewertet.

**Ergebnisse:**

Die Interventionsgruppe zeigte im Laufe der Zeit eine signifikante Verringerung der krankheitsbezogenen Fehlannahmen (p < 0,001; η_p_^2^ = 0,12) und Zustimmung zu Stereotypen (p* = *0,002; η_p_^2^ = 0,12). In beiden Gruppen nahm der Wunsch nach sozialer Distanz nach dem Seminar ab, erreichte bei der Nachuntersuchung jedoch wieder den Ausgangswert.

**Schlussfolgerungen:**

Das Seminar hat die stigmatisierenden Überzeugungen und Einstellungen von FGKP in Bezug auf Hauterkrankungen verbessert. Seine Integration in Berufsbildungslehrpläne oder die Durchführung in Workshops, um das Wissen über Hauterkrankungen zu erweitern und Vorurteile in verschiedenen Berufsgruppen abzubauen, ist vielversprechend.

## EINLEITUNG

Menschen mit sichtbaren chronischen Hauterkrankungen sind häufig Stigmatisierung ausgesetzt. Mehrere Umfragen ergaben, dass die Mehrheit der Menschen mit Psoriasis angab, bereits diskriminiert oder gedemütigt worden zu sein,^1^, ^2^, ^3^ was mit den Erkenntnissen übereinstimmt, dass andere Menschen den Körperkontakt meiden,^4^ und dass die Krankheit Beziehungen beeinträchtigt.^5^ Ebenso berichten Menschen mit anderen Hauterkrankungen wie atopischer Dermatitis, Akne inversa und anderen ebenfalls, dass sie angewiderte Blicke erfahren haben (20,6 %), dass ihnen der Zugang zu Freizeiteinrichtungen verwehrt wurde (11,6 %) und dass sie sozial ausgegrenzt wurden (15,6 %)^6^. Studien aus den USA und Deutschland, in denen die Einstellung der Allgemeinbevölkerung gegenüber Menschen mit chronischen Hauterkrankungen untersucht wurde, bestätigen dieses Bild.^7^, ^8^, ^9^ Für Menschen mit chronischen Hauterkrankungen wirken sich diese Erfahrungen negativ auf ihr psychisches Wohlbefinden, ihre sozialen Interaktionen und ihre Lebensqualität aus.^1^, ^10^, ^11^, ^12^, ^13^

Diese erheblichen Auswirkungen auf die Betroffenen unterstreichen die Notwendigkeit, das Stigma in der Öffentlichkeit zu bekämpfen. In einer systematischen Übersicht identifizierten Topp et al.^14^ nur neun Studien von eher geringer Qualität, in denen Ansätze zur Verringerung des öffentlichen Stigmas getestet wurden, welche sich alle auf Lepra bezogen. Daher besteht ein dringender Bedarf an der Entwicklung und gründlichen Erprobung von Ansätzen zur Verringerung des Stigmas auch im Zusammenhang mit anderen Hauterkrankungen. In ihrer Resolution von 2014 forderte die Weltgesundheitsversammlung (WHA) ihre Mitgliedstaaten auf, Maßnahmen zu ergreifen, um die Akzeptanz von Menschen mit Psoriasis zu erhöhen.^15^ Als Reaktion darauf wurde in Deutschland das Projekt ECHT,^16^ durchgeführt, das darauf abzielte, chronische Hauterkrankungen unter Medizinstudenden,^17^ und Erziehern in der Ausbildung,^18^ zu entstigmatisieren. Die Teilnehmer nahmen an einem Seminar teil, das Selbstreflexionsübungen, einen theoretischen Vortrag über Hauterkrankungen und Stigmatisierung sowie eine Begegnung mit einem Patienten umfasste. Diese Interventionsstruktur entspricht der Empfehlung von Heijnders und van der Meij,^19^ bei der Bekämpfung von Stigmatisierung auf Gesellschaftsebene Bildung mit einem Kontaktansatz zu kombinieren, und erwies sich in der Tat als erfolgreich bei der Verringerung stigmatisierender Überzeugungen und Einstellungen gegenüber Menschen mit chronischen Hauterkrankungen.^17^, ^18^

Ein weiterer Bereich, in dem Menschen mit chronischen Hauterkrankungen Stigmatisierung erfahren, ist die Gesundheits‐ und Körperpflege: Fast 10 % der Befragten der Clear‐About‐Psoriasis‐Umfrage,^1^ wurde eine Haarschnitt‐ oder Kosmetikbehandlung verweigert. Ähnliche Zahlen wurden bereits vor 25 Jahren beobachtet.^20^ Friseure, Kosmetikerinnen, Physiotherapeuten und Pflegekräfte haben physischen Kontakt mit ihren Kunden, und sowohl ihre offene Ablehnung als auch subtilere Zurückhaltung und Mimik als Reaktion auf Hauterkrankungen können einen erheblichen Einfluss auf das Wohlbefinden der Patienten und ihre Bereitschaft, diese Dienstleistungen in Anspruch zu nehmen, haben. Daher zielte dieses Projekt darauf ab, die „ECHT“‐Intervention für Berufe im Bereich der Körperpflege anzupassen und ihre Wirksamkeit zu bewerten. Es wurde die Hypothese aufgestellt, dass die Interventionsgruppe im Vergleich zur Kontrollgruppe eine stärkere Verringerung der stigmatisierenden Einstellungen gegenüber Menschen mit chronischen Hautkrankheiten zeigen würde.

## MATERIALIEN UND METHODIK

### Teilnehmer

Die Teilnehmer wurden über Mailinglisten und Newsletter von Berufsschulen, Berufsverbänden und dem örtlichen Universitätsklinikum, über Beiträge in sozialen Medien sowie mittels Flyer in örtlichen Friseur‐ und Kosmetiksalons rekrutiert. Basierend auf einer A‐priori‐Berechnung der Stichprobengröße mit G*Power,^21^ (F‐Tests für eine zweifaktorielle Varianzanalyse mit Messwiederholung) mit der von Weinberger et al.^18^ ermittelten Effektgröße (Interaktionseffekt für Verhaltensabsichten: η_p_^2^ = 0,02) sind 82 Teilnehmer erforderlich, um eine Aussagekraft von 0,80 (α = 0,05) zu erreichen. Um Mehrfachprüfungen und eine potenzielle Dropout‐Rate von bis zu 20 % bei der Nachuntersuchung zu berücksichtigen, wurde eine Gesamtteilnehmerzahl von 120 Personen angestrebt.

Die selbstberichteten Einschlusskriterien umfassten: Mindestalter 18 Jahre; Tätigkeit als Friseurin, Kosmetikerin, Pflegekraft oder Physiotherapeut oder mindestens im zweiten Ausbildungsjahr für einen dieser Berufe; Fähigkeit zur selbstständigen Einwilligung sowie ausreichende Beherrschung der deutschen Sprache.

Die Teilnehmer gaben zu Beginn des Seminars ihre schriftliche Einwilligung und erhielten eine finanzielle Entschädigung für die Seminarteilnahme (40 €) und das Ausfüllen des Follow‐up‐Fragebogens (10 €). Die Studie wurde in Übereinstimmung mit der Helsinki‐Erklärung durchgeführt und von der Ethikkommission des Universitätsklinikums Hamburg‐Eppendorf genehmigt (Genehmigungsnummer: LPEK‐0395).

### Vorgehen

Die an der Teilnahme interessierten Personen registrierten sich über die Studienwebsite für einen der angebotenen Seminartermine. Aus Gründen der Durchführbarkeit wurde eine Randomisierung vorgenommen, bei der die Seminartermine nach dem Zufallsprinzip entweder den Interventions‐ oder den Kontrollseminaren zugeordnet wurden.

Die Teilnehmer nahmen an einem Präsenzseminar am Universitätsklinikum Hamburg‐Eppendorf teil. Bei ihrer Ankunft lasen die Teilnehmer die Teilnahmeinformationen und unterzeichneten die Einverständniserklärung. Anschließend füllten sie den Vorab‐Fragebogen (t0) aus, gefolgt von einer kurzen Vorstellungsrunde. Anschließend wurde das Interventions‐ oder Kontrollprogramm (beide 2,5 Stunden lang; Details siehe unten) von einer der Autorinnen (RS, JT) durchgeführt. Nach Abschluss füllten die Teilnehmer den Post‐Test‐Fragebogen (t1) aus. Drei Monate später erhielten die Teilnehmer per E‐Mail den Link zum Follow‐up‐Fragebogen (t2).

*Interventionsgruppe*: In der Interventionsgruppe erhielten die Teilnehmer eine modifizierte Version des „ECHT“‐Seminars,^17^, ^18^ das aus drei Komponenten bestand: *(1)* drei Selbstreflexionsübungen zur eigenen Anfälligkeit für Stigmatisierung, zum Stigmatisierungsrisiko bei 24 verschiedenen Hauterkrankungen und zur Reflexion darüber, mit welcher Hauterkrankung die Teilnehmer am besten und am wenigsten leben könnten (30 Min.); *(2)* einen Vortrag über Hauterkrankungen und Stigmatisierung mit Fallbeispielen zu Urtikaria und Psoriasis (20 Min.); und *(3)* eine Begegnung mit einer Person mit Psoriasis, die ihre persönlichen Erfahrungen mit dem Leben mit einer chronischen Hauterkrankung mit Schwerpunkt auf Stigmatisierung teilte und Fragen der Teilnehmer beantwortete (60 Min.). Abgesehen von der Begegnung mit dem Patienten waren die Seminarinhalte nicht erkrankungsspezifisch.

*Kontrollgruppe*: Die Kontrollgruppe erhielt ein Seminar, das in Struktur und Grad der erforderlichen Beteiligung/Aktivität mit dem der Interventionsgruppe vergleichbar war, sich jedoch auf die Gesundheit der Teilnehmer am Arbeitsplatz konzentrierte und aus folgenden Teilen bestand: *(1)* einer Selbstreflexionsphase, in der die Teilnehmer über ihre eigenen Erfahrungen mit physischem und psychischem Stress am Arbeitsplatz reflektierten (30 Min.); *(2)* einen Vortrag zum Thema Stress und Stressbewältigungstechniken (20 Min.); und *(3)* ein E‐Learning‐Tool zum Thema psychische Gesundheit für Arbeitnehmende (60 Min.).

### Endpunkte

Die folgenden selbstberichteten Endpunkte wurden über die Online‐Plattform Unipark (http://www.unipark.com) oder auf Papier erfasst.

#### Primäre Endpunkte

Die Instrumente zur Bewertung der Stigmatisierung wurden von Pearl et al.^9^ angepasst und entwickelt und von Sommer et al.^17^ ins Deutsche übersetzt. Diese Instrumente konzentrierten sich beispielhaft auf Psoriasis.

*Zustimmung zu negativen Stereotypen*: Die Zustimmung zu negativen Stereotypen über Menschen mit Psoriasis wurde anhand einer Skala bewertet, die aus elf Adjektivpaaren bestand (zum Beispiel unattraktiv – attraktiv). Die Teilnehmer wurden gebeten, den Kreis zu markieren, der dem Adjektiv am nächsten kam, welches ihrer Meinung nach eine Person mit Psoriasis am besten beschrieb (Skala: 1–5). Die Werte wurden gemittelt, wobei höhere Werte eine stärkere Zustimmung zu negativen Stereotypen widerspiegelten. Die interne Konsistenz in dieser Studie war zu allen Zeitpunkten gut (alle α ≥ 0,83).

*Krankheitsbezogene Fehlannahmen*: Um die Zustimmung zu verbreiteten falschen Vorstellungen über Psoriasis zu messen, gaben die Teilnehmer ihren Grad der Zustimmung zu 15 Aussagen (zum Beispiel „Psoriasis ist ansteckend“) auf einer 5‐Punkte‐Likert‐Skala von „stimme überhaupt nicht zu“ bis „stimme voll und ganz zu“ an. Die Werte wurden gemittelt, wobei höhere Werte eine stärkere Zustimmung zu diesen Fehlannahmen widerspiegelten. Die interne Konsistenz innerhalb dieser Studie war zu allen Zeitpunkten akzeptabel (alle α ≥ 0,65).

*Wunsch nach sozialer Distanz*: Die Teilnehmer bewerteten ihren Wunsch nach sozialer Distanz gegenüber Menschen mit Psoriasis in neun Situ ationen (zum Beispiel Händeschütteln) auf einer 5‐Punkte‐Likert‐Skala von „auf keinen Fall“ bis „auf jeden Fall“. Die Werte wurden gemittelt, wobei höhere Werte einen stärkeren Wunsch nach Distanz widerspiegelten. Die interne Konsistenz innerhalb dieser Studie war zu allen Zeitpunkten gut (alle α ≥ 0,86).

*Berichtetes und beabsichtigtes Verhalten*: Die von Evans‐Lacko et al.^28^ entwickelte Skala für berichtetes und beabsichtigtes Verhalten besteht aus vier Items, die das Vorhandensein von Verhalten (ja/nein) in jedem von vier Kontexten (Zusammenleben mit, Arbeiten mit, Wohnen in der Nähe von und Fortsetzung einer Beziehung mit jemandem, der von einer chronischen Hautkrankheit betroffen ist) und vier weiteren Items, welche die Verhaltensabsichten in diesen Kontexten in der Zukunft bewerten. Die Teilnehmer bewerten jedes Item auf einer 5‐Punkte‐Likert‐Skala von „stimme überhaupt nicht zu“ bis „stimme voll und ganz zu“. Es wurde eine Gesamtpunktzahl für die vier Items zum „künftigen Verhalten“ berechnet, wobei niedrigere Punktzahlen auf ein stärker stigmatisierendes Verhalten hindeuten. Die interne Konsistenz war in dieser Studie zu allen Zeitpunkten gut (alle α ≥ 0,81).

#### Sekundäre Endpunkte

*Zufriedenheit mit dem Seminar*: Die Zufriedenheit der Teilnehmer mit dem Seminar wurde zu Zeitpunkt t1 anhand von zwölf Fragen bewertet, die sich auf die allgemeine Zufriedenheit mit dem Seminar und dessen Umfang, die persönliche und berufliche Relevanz, die Vorbereitung auf ähnliche Situationen am Arbeitsplatz und die Bereitschaft, das Seminar einem Kollegen weiterzuempfehlen, bezogen.^17^, ^18^ Die Aussagen wurden auf einer 5‐Punkte‐Likert‐Skala bewertet, die von „stimme voll zu” bis „stimme überhaupt nicht zu” reichte, wobei niedrigere Werte eine höhere Zufriedenheit anzeigten. Weitere sekundäre Endpunkte sind im Zusatzmaterial (S1) beschrieben.

### Statistische Analysen

Die statistischen Analysen wurden von einer verblindeten Analystin (CBvS) unter Verwendung des Statistical Package for the Social Sciences (SPSS v. 27; IBM Corp., Armonk, NY, USA) durchgeführt. Es wurden deskriptive Statistiken für soziodemografische Daten, eigene Hauterkrankungen, Berufserfahrung und Häufigkeit des Kontakts mit betroffenen Personen erstellt. Unterschiede zwischen den Gruppen wurden mit Hilfe von t‐Tests für unabhängige Stichproben, Mann‐Whitney‐U‐Tests und χ^2^‐Tests analysiert. Annahmen der Normalität und Sphärizität wurden mit Hilfe des Shapiro‐Wilk‐Tests beziehungsweise des Mauchly‐Tests auf Sphärizität überprüft.

Die Effekte der Intervention auf die primären Endpunkte wurden mittels 2 (Gruppe: Intervention, Kontrolle) x 3 (Zeit: t0, t1, t2) Varianzanalysen mit Messwiederholung (ANOVA) analysiert. Es wurden Subgruppenanalysen durchgeführt, um mögliche Unterschiede zwischen den Berufsgruppen zu bewerten. Aufgrund der geringen Stichprobengröße bei Friseurinnen und Kosmetikern, aber vergleichbarer Berufsausbildung, wurden diese Berufe in einer Beauty‐Gruppe zusammengefasst. Die Auswirkungen der Intervention auf die primären Ergebnisse nach Beruf wurden mittels 2 (Gruppe: Intervention, Kontrolle) x 3 (Zeitpunkt: t0, t1, t2) x 3 (Beruf: Beauty, Krankenschwestern, Physiotherapeuten) ANOVAs mit Messwiederholung analysiert. Für die Variable „Wunsch nach sozialer Distanz“ wurde die Annahme der Sphärizität verletzt (p* = *0,001), weshalb Huynh‐Feldt‐korrigierte Ergebnisse angegeben werden. Die p‐Werte wurden aus zweiseitigen Tests ermittelt, wobei p* < *0,05 auf statistische Signifikanz hinweist. Es wurde eine Bonferroni‐Korrektur für multiple Tests angewendet. Die Effektgrößen werden als η_p_^2^ angegeben, wobei η_p_^2^ < 0,06 eine kleine Effektstärke, η_p_^2^≥ 0,06 eine mittlere Effektstärke und η_p_^2^ ≥ 0,14 eine große Effektstärke darstellt.^29^ Signifikante Effekte wurden anhand eines Post‐hoc‐Paarvergleichs genauer untersucht.

### Ethik

Das Forschungsvorhaben wurde von der lokalen Ethikkommission des Universitätsklinikums Hamburg‐Eppendorf geprüft und genehmigt (#LPEK‐0395). Die Teilnehmer dieser Studie haben ihre schriftliche Einwilligung zur Veröffentlichung ihrer anonymisierten Daten gegeben.

### Erklärung zur Datenverfügbarkeit

Das Studienprotokoll, die Interventionsmaterialien und der Analysecode sind zu finden unter: https://osf.io/zaqg6.

Die Daten der individuellen Teilnehmer, die den in diesem Artikel berichteten Ergebnissen zugrunde liegen, sind nach Anonymisierung (Text, Tabellen, Abbildungen und Anhänge) auf begründete Anfrage von der korrespondierenden Autorin erhältlich, und zwar beginnend 3 Monate und endend 5 Jahre nach Veröffentlichung des Artikels. Die Daten können an Wissenschaftler weitergegeben werden, deren vorgeschlagene Verwendung der Daten von einem unabhängigen Prüfungsausschuss (learned intermediary) genehmigt wurde, der zu diesem Zweck benannt wurde.

## ERGEBNISSE

### Stichprobenbeschreibung

**ABBILDUNG 1:**
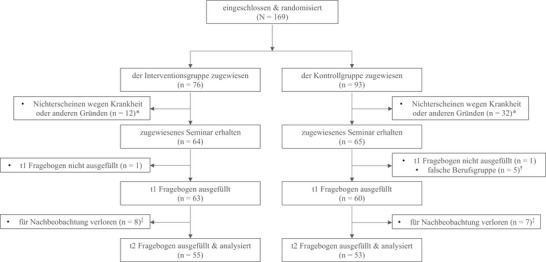
Teilnehmerflussdiagramm. *Zu diesem Zeitpunkt wussten die Teilnehmer noch nicht, welcher Gruppe sie zugeordnet worden waren. ^†^Diese Teilnehmer gaben während des Seminars an, dass sie nicht zu einer der Zielberufsgruppen gehörten. ^‡^Diese Teilnehmer reagierten weder auf die E‐Mail‐Einladung noch auf die Erinnerung zur Teilnahme an der t2‐Erhebung; Gründe für die Nichtbeantwortung konnten nicht ermittelt werden.

Zwischen Oktober 2022 und Februar 2023 wurden 15 Seminare durchgeführt, davon sieben für die Interventionsgruppe und acht für die Kontrollgruppe, um eine ungefähr gleiche Teilnehmerzahl in beiden Gruppen zu erreichen. Alle Seminare begannen um 18:00 Uhr oder 18:30 Uhr an einem Wochentag (Montag bis Donnerstag). Insgesamt nahmen 129 Teilnehmer an diesen Seminaren teil, davon 64 in der Interventionsgruppe und 65 in der Kontrollgruppe. Im Durchschnitt nahmen 8,6 Teilnehmer an jedem Seminar teil. Vollständige Datensätze für alle drei Zeitpunkte lagen von 108 Teilnehmer vor (t2‐Rücklaufquote: 90 %), davon 55 in der Interventionsgruppe und 53 in der Kontrollgruppe (Abbildung 1). Die demografischen Daten dieser Teilnehmer sind in Tabelle 1 dargestellt. Die beiden Gruppen waren hinsichtlich aller demografischen Variablen und Ausgangswerte der primären Endpunkte vergleichbar. Die Mehrheit der Teilnehmer (63 %) war hinsichtlich ihrer Gruppenzugehörigkeit verblindet, wobei es keinen Unterschied zwischen den beiden Gruppen gab. Allerdings waren nur 55,6 % bezüglich des Studienziels verblindet, wobei in der Kontrollgruppe deutlich mehr Personen verblindet blieben (69,8 %) als in der Interventionsgruppe (41,8 %; p < 0,001).

**TABELLE 1:** Deskriptive Statistik der soziodemografischen Daten und Ausgangswerte der primären Endpunkte.

		Gesamt (n = 108)	Intervention (n = 55)	Kontrolle (n = 53)
Geschlecht	Weiblich	95 (88%)	49 (89,1%)	46 (86.8%)
	Männlich	12 (11,1%)	5 (9,1%)	7 (13.2%)
	Divers	1 (0,9%)	1 (1,8%)	–
Alter, in Jahre		32,12 ± 12,88	30,53 ± 12,62	33,67 ± 13,08
Beruf	Beauty	18 (16,6%)	10 (18,2%)	8 (15,1%)
	Pflegekraft	41 (38,0%)	22 (40,0%)	19 (35,8%)
	Physiotherapie	49 (45,4%)	23 (41,8%)	26 (49,1%)
Berufserfahrung, in Jahren		9,04 ± 11,19	7,86 ± 10,54	10,28 ± 11,82
Von einer Hauterkrankung betroffen		18 (16,7%)	10 (18,2%)	8 (15,1%)
Häufigkeit des Kontakts mit Personen, die von einer Hauterkrankung betroffen sind	Nie	4 (3,7%)	3 (5,5%)	1 (1,9%)
	Selten	30 (27,8%)	13 (23,6%)	17 (32,1%)
	Gelegentlich	38 (35,2%)	22 (40,0%)	16 (30,2%)
	Oft	26 (24,1%)	11 (20,0%)	15 (28,3%)
	Täglich	7 (6,5%)	4 (7,3%)	3 (5,7%)
	Fehlend	2 (1,9%)	1 (1,8%)	1 (1,9%)
Verblindet hinsichtlich des Studienziels (%)		60 (55,6%)	23 (41,8%)	37 (69,8%)
Einschätzung der Gruppenzugehörigkeit	Korrekt	38 (35,2%)	18 (32,7%)	20 (37,7%)
	Inkorrekt	68 (63,0%)	36 (65,5%)	32 (60,4%)
	Unsicher	2 (1,9%)	1 (1,8%)	1 (1,9%)
Zustimmung zu negativen Stereotypen		2,75 ± 0,62	2,73 ± 0,63	2,77 ± 0,61
Wunsch nach sozialer Distanz		1,67 ± 0,54	1,60 ± 0,49	1,75 ± 0,58
Krankheitsbezogene Fehlannahmen		2,06 ± 0,43	2,08 ± 0,41	2,07 ± 0,45
Stigmatisierendes Verhalten		18,22 ± 2,50	18,43 ± 2,32	17,98 ± 2,68

*Anmerkung*: Beauty: Friseurinnen und Kosmetiker

### Primäre Endpunkte

**ABBILDUNG 2:**
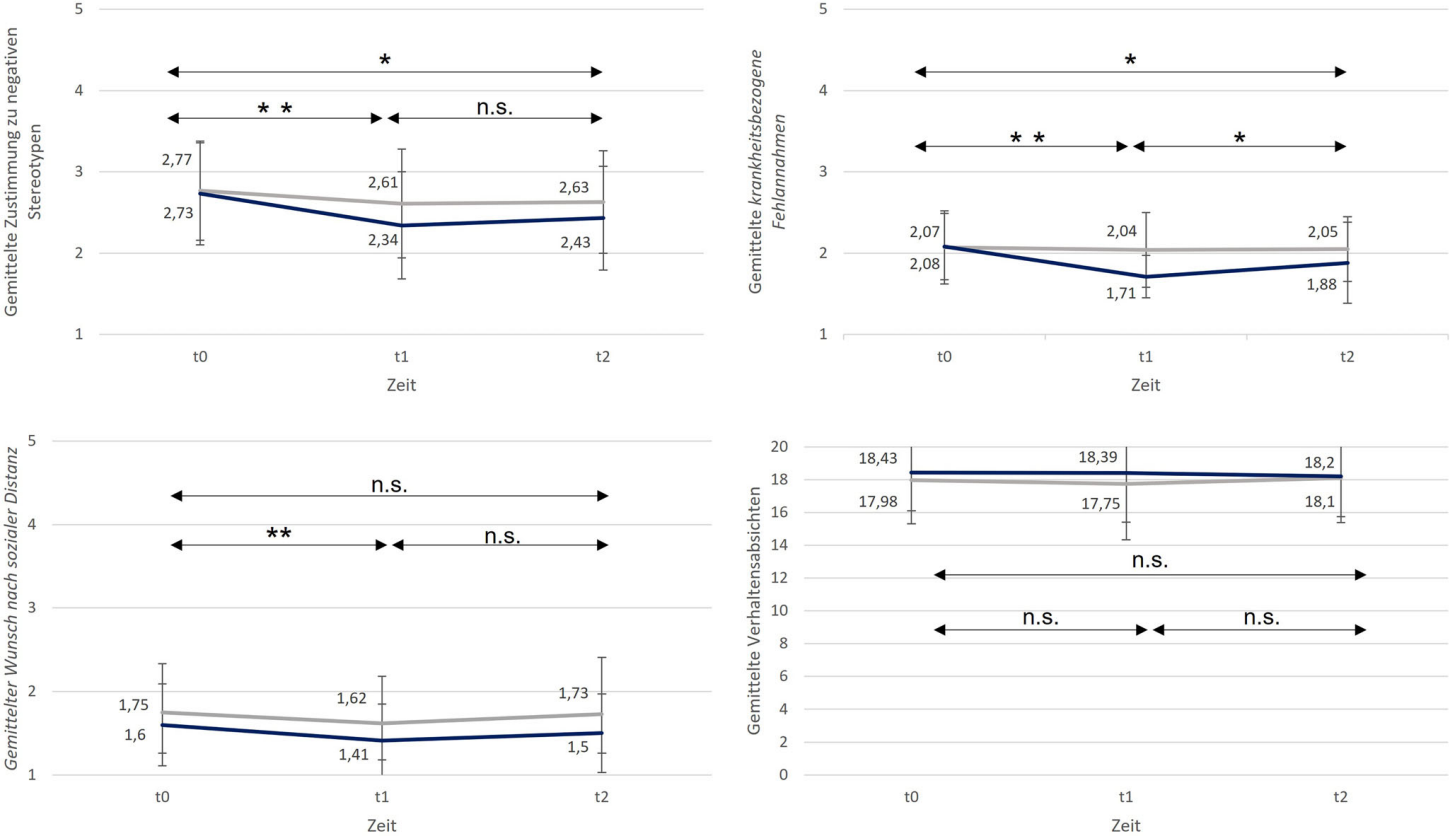
Mittelwerte der primären Endpunkte für die Interventions‐ und Kontrollgruppe zu t0 (vor dem Test), t1 (nach dem Test) und t2 (3‐Monats‐Nachbeobachtung). n.s., nicht signifikant; *p < 0,05; **p < 0,001.

Die Abbildung 2 zeigt die Veränderungen der primären Endpunkte im Zeitverlauf für die Interventions‐ und die Kontrollgruppe. Die Tabelle 2 enthält die Mittelwerte und Standardabweichungen (SD) der primären Endpunkte zu t0, t1 und t2.

**TABELLE 2:** Deskriptive Statistik (Mittelwert [MW], Standardabweichung [SD]) der primären Endpunkte zu t0 (vor dem Test), t1 (nach dem Test) und t2 (Nachuntersuchung) sowie gruppenspezifische Veränderungen im Zeitverlauf.

	Intervention (n = 55)			Kontrolle (n = 53)		
	*Mittelwert (SD)*	*Vergleich*	*Differenz*	*Mittelwert (SD)*	*Vergleich*	*Differenz*
***Zustimmung zu negativen Stereotypen***						
t0	2,73 (0,63)	t1–t0	p* < *0,001	2,77 (0,61)	t1–t0	p* = *0,10
t1	2,34 (0,66)	t2–t0	p* = *0,002	2,61 (0,67)	t2–t0	p* = *0,33
t2	2,43 (0,64)	t2–t1	p* = *0,69	2,63 (0,63)	t2–t1	p > 0,99
***Wunsch nach sozialer Distanz***						
t0	1,60 (0,49)	t1–t0	p* < *0,001	1,75 (0,58)	t1–t0	p* = *0,013
t1	1,41 (0,44)	t2–t0	p* = *0,10	1,62 (0,56)	t2–t0	p* = *0,81
t2	1,50 (0,47)	t2–t1	p* = *0,16	1,74 (0,68)	t2–t1	p* = *0,033
***Krankheitsbezogene Fehlannahmen***						
t0	2,08 (0,41)	t1–t0	p* < *0,001	2,07 (0,45)	t1–t0	p > 0,99
t1	1,71 (0,26)	t2–t0	p* < *0,001	2,04 (0,46)	t2–t0	p > 0,99
t2	1,88 (0,50)	t2–t1	p* = *0,003	2,05 (0,40)	t2–t1	p > 0,99
***Verhaltensabsichten***						
t0	18,43 (2,68)	t1–t0	p > 0,99	17,98 (2,32)	t1–t0	p > 0,99
t1	18,39 (3,42)	t2–t0	p > 0,99	17,75 (2,99)	t2–t0	p > 0,99
t2	18,20 (2,71)	t2–t1	p > 0,99	18,10 (2,71)	t2–t1	p* = *0,97

*Zustimmung zu negativen Stereotypen*: Es zeigte sich ein signifikanter Haupteffekt der Zeit auf die Zustimmung zu negativen Stereotypen, F(2,212) = 13,74, p < 0,001, η_p_^2^ = 0,12. Es wurde weder ein Haupteffekt der Gruppe, F(1,106) = 2,65, p = 0,22, noch ein Interaktionseffekt, F(2,212) = 2,30, p = 0,10, festgestellt. Post‐hoc‐Tests zeigten, dass in der Interventionsgruppe die Zustimmung zu negativen Stereotypen von t0 zu t1 (Differenz_t0–t1_: 0,392, p < 0,001, 95 %‐Konfidenzintervall [KI] 0,22–0,57) und t2 (Differenz_t0–t2_: 0,299, p = 0,002, 95 %‐KI 0,1–0,5) signifikant abnahm, und zwischen t1 und t2 stabil blieb (Differenz_t1–t2_: –0,093, p = 0,69, 95 %‐KI –0,28–0,09). In der Kontrollgruppe gab es keinen signifikanten Unterschied zwischen den drei Zeitpunkten.

*Krankheitsbezogene Fehlannahmen*: Es zeigten sich signifikante Haupteffekte von Zeit, F(2,210) = 20,50, p < 0,001, η_p_^2^ = 0,16, und Gruppe, F(1,105) = 4,52, p = 0,036, η_p_^2^ = 0,04, in Bezug auf krankheitsbezogene Fehlannahmen sowie ein signifikanter Interaktionseffekt, F(2,210) = 14,81, p < 0,001, η_p_^2^ = 0,12. Post‐hoc‐Tests zeigten, dass in der Interventionsgruppe die krankheitsbezogenen Fehlannahmen von t0 zu t1 (Differenz_t0–t1_: 0,398, p < 0,001, 95 %‐KI 0,28–0,51) und t2 (Differenz_t0–t2_: 0,225, p < 0,001, 95 %‐KI 0,12–0,33) signifikant abnahmen, aber von t1 zu t2 wieder zunahmen [Differenz_t1–t2_: ‐0,173, p = 0,003, 95 %‐KI –0,30–(–0,05)]. In der Kontrollgruppe gab es keine signifikanten Unterschiede zwischen den drei Zeitpunkten.

*Wunsch nach sozialer Distanz*: In Bezug auf den Wunsch nach sozialer Distanz gegenüber Menschen mit Psoriasis wurden signifikante Haupteffekte von Zeit, Huynh‐Feldt F(1,83,194,26) = 8,53, p < 0,001, η_p_^2^ = 0,074, und Gruppe, F(1,106) = 4,41, p = 0,038, η_p_^2^ = 0,040, festgestellt. Der Interaktionseffekt, Huynh‐Feldt F(1,83,194,26) = 0,544, p = 0,57, war nicht signifikant. Post‐hoc‐Tests zeigten, dass der Wunsch nach sozialer Distanz sowohl in der Interventionsgruppe (Differenz_t0–t1_: 0,186, p < 0,001, 95 %‐KI 0,08–0,29) als auch in der Kontrollgruppe (Differenz_t0–t1_: 0,135, p = 0,012, 95 %‐KI 0,02–0,25) signifikant abnahm. Allerdings erreichten die Werte in beiden Gruppen zu t2 fast wieder den Ausgangswert (Interventionsgruppe: Differenz_t0–t2_: 0,097, p = 0,35, 95 %‐KI –0,05–0,25; Kontrollgruppe: Differenz_t0–t2_: 0,016, p > 0,99, 95 %‐KI –0,136–0,168).

Berichtetes und beabsichtigtes Verhalten. Es wurde kein signifikanter Haupteffekt von Zeit, F(2,208) = 0,18, p = 0,84, oder Gruppe, F(1,104) = 0,70, p = 0,41, auf die Verhaltensabsichten festgestellt, und auch der Interaktionsterm war nicht signifikant, F(2,208) = 0,72, p = 0,49.

### Sekundäre Endpunkte

Die Zufriedenheit mit dem Seminar und dessen wahrgenommene Relevanz waren im Allgemeinen hoch. Im Vergleich zur Kontrollgruppe waren die Teilnehmer der Interventionsgruppe jedoch insgesamt deutlich zufriedener mit dem Seminar (Kontrolle: Mittelwert (MW) = 4,45, SD = 0,70; Intervention: MW = 4,78, SD = 0,69), t(105,72) = –2,48, p = 0,015, fühlten sich besser auf die Praxis vorbereitet (Kontrolle: MW = 3,79, SD = 0,72; Intervention: MW = 4,29, SD = 1,12), t(92,49)=‐2,77, p = 0,007, und waren eher bereit, das Seminar ihren Kollegen weiterzuempfehlen (Kontrolle: MW = 4,60, SD = 0,60; Intervention: MW = 4,84, SD = 0,46), t(97,74)=‐2,25, p = 0,027.

Die Ergebnisse der Subgruppenanalysen nach Beruf, Analysen unter Berücksichtigung früherer Erfahrungen mit Hauterkrankungen sowie Ergebnisse in Bezug auf Feuermale und zu einem zusätzlichen sekundären Endpunkt (emotionale Reaktionen auf Bilder von Hauterkrankungen) sind im Online‐Supplement (S2–S5) zu finden.

## DISKUSSION

Basierend auf dem Aufruf der WHA^15^ zur Bekämpfung von Stigmatisierung und dem ECHT‐Projekt,^17^, ^18^ evaluierte diese Studie die Wirksamkeit eines Gruppenseminars zur Verringerung stigmatisierender Einstellungen und zur Verbesserung der Verhaltensabsichten gegenüber chronischen Hauterkrankungen bei Fachkräften im Gesundheits‐ und Körperpflegebereich. Die Ergebnisse bestätigten einen signifikanten Rückgang stigmatisierender Einstellungen, der teilweise auf das Seminar zurückgeführt werden kann: Insbesondere hatte die Interventionsgruppe nach der Teilnahme am Seminar deutlich weniger krankheitsbezogene Fehlannahmen als die Kontrollgruppe. Die Interventionsgruppe zeigte auch sowohl beim Post‐Test als auch bei der Nachuntersuchung nach 3 Monaten eine signifikante Verringerung der Zustimmung zu negativen Stereotypen, obwohl sich diese Veränderungen im Vergleich zur Kontrollgruppe nicht signifikant unterschieden. Überraschenderweise nahm die soziale Distanz in beiden Gruppen vom Ausgangswert bis zum Post‐Test signifikant ab, aber diese Effekte verschwanden bei der Nachuntersuchung. Wichtig ist, dass krankheitsbezogene Fehlannahmen, nicht jedoch die Zustimmung zu Stereotypen oder der Wunsch nach sozialer Distanz gegenüber Menschen mit Feuermalen, im Laufe der Zeit in der Interventionsgruppe ebenfalls abnahmen (Online‐Supplement S4). Dies deutet darauf hin, dass sich die Auswirkungen des Seminars auch auf andere Hauterkrankungen übertragen lassen, die in der Vorlesung oder bei der Begegnung mit Patienten nur in geringem Umfang behandelt wurden. Bei beiden Gruppen wurden keine Veränderungen hinsichtlich der Verhaltensabsichten gegenüber Menschen mit chronischen Hauterkrankungen beobachtet. Das Seminar schien für die verschiedenen Berufsgruppen gleichermaßen geeignet zu sein (Online‐Supplement S2).

Die Ergebnisse stimmen weitgehend mit früheren Studien überein: Sowohl Medizinstudenten und angehende Pädagogen in der ECHT‐Studie als auch Personen aus der Allgemeinbevölkerung, die an einem Programm zur Stigmatisierungsreduktion auf Instagram teilnahmen,^22^ berichteten nach der Intervention von weniger krankheitsbezogenen Fehlannahmen und einer geringeren Zustimmung zu negativen Stereotypen. Allerdings zeigten nur die ECHT‐Teilnehmer geringe Veränderungen in ihren Verhaltensabsichten, die über einen Zeitraum von drei Monaten nicht anhielten. Dies könnte mehrere Gründe haben: Von Anfang an waren die selbst‐berichteten Verhaltensabsichten gegenüber Menschen mit Psoriasis insbesondere in der aktuellen Stichprobe sehr positiv, sodass möglicherweise ein Deckeneffekt eingetreten ist. Darüber hinaus gilt Wissen zwar als Voraussetzung für eine Änderung der Einstellung und des Verhaltens,^10^, ^25^, ^26^ und mediiert nachweislich die Auswirkungen des Kontakts mit Mitgliedern einer stigmatisierten Gruppe auf vorurteilsbehaftete Einstellungen,^30^ doch spielen auch andere Faktoren wie Motivation eine Rolle.^23^ Die ECHT‐Seminare wurden im Rahmen des Studiums und der Berufsausbildung der Teilnehmer durchgeführt, was trotz ihres freiwilligen Charakters die Teilnehmer möglicherweise motiviert hat, sich noch stärker zu engagieren und zu lernen. Darüber hinaus erhielt die Kontrollgruppe in dieser Studie im Gegensatz zur Kontrollgruppe im ECHT‐Projekt, die sich mit dem Lesen dermatologischer Fachartikel befasste, ein Seminar, das in Bezug auf Struktur, Dauer und Grad der erforderlichen Beteiligung/Aktivität dem der Interventionsgruppe ähnelte, was möglicherweise zu den geringeren Effekten auf die Verhaltensabsichten beigetragen hat.

Was die Verallgemeinerbarkeit der vorliegenden Ergebnisse angeht, so bestand die Mehrheit der Stichprobe aus Frauen (88 %), was etwas über dem liegt, was aufgrund der Geschlechterverteilung in den Zielberufen zu erwarten wäre.^28^, ^29^, ^30^ Das Alter der Teilnehmer deckte jedoch den Bereich der regulären Erwerbsbevölkerung ab (20–66 Jahre) und sie stammten aus unterschiedlichen sozioökonomischen Verhältnissen (59,3 % der Stichprobe gaben ein monatliches Haushaltseinkommen von 2000 € oder weniger an, 14 % gaben ein Einkommen von > 4000 € an; 60 % hatten das (Fach‐)Abitur, 7 % einen Hochschulabschluss). Daher sind unsere Ergebnisse auf die breitere Zielgruppe übertragbar.

### Limitationen und Ausblick

Bei der Interpretation der Ergebnisse dieser Studie sind mehrere Limitationen zu berücksichtigen: Erstens war die Verblindung der Teilnehmer nicht vollständig erfolgreich. Obwohl fast zwei Drittel der Teilnehmer keine Kenntnis über ihre Gruppenzugehörigkeit hatten, blieb nur die Hälfte der Stichprobe über das Ziel der Studie im Unklaren. Dies könnte daran liegen, dass sich die Fragebögen stark auf die Einstellung gegenüber Hauterkrankungen konzentrierten und somit sozial erwünschte Antworten begünstigt haben könnten. Darüber hinaus waren viele Teilnehmer Klassenkameraden oder Kollegen und könnten trotz der Anweisung, dies nicht zu tun, möglicherweise ihre Erfahrungen miteinander geteilt haben, sodass die Teilnehmer auch etwas über den Inhalt des anderen Seminars erfahren haben. Eine weitere Einschränkung ist die relativ kurze Nachbeobachtungszeit von 3 Monaten.

Zukünftige Studien sollten die längerfristigen Effekte untersuchen, Verhaltensmaßnahmen einsetzen, um das Problem der sozialen Erwünschtheit zu umgehen, und die Einstellung gegenüber anderen Hauterkrankungen erfassen. Darüber hinaus könnten die Identifizierung der aktiven Elemente des Seminars, um es möglicherweise zu verkürzen, die Untersuchung der Wirksamkeit einer Online‐Version des Seminars und die Erprobung des Nutzens einer Auffrischungssitzung dazu beitragen, die Ergebnisse zu verbessern und gleichzeitig die Integration in Berufsbildungslehrpläne zu erleichtern.

### Schlussfolgerungen

Das Seminar trug erfolgreich dazu bei, stigmatisierende Vorurteile gegenüber chronischen Hauterkrankungen abzubauen, jedoch nicht stigmatisierende Verhaltensweisen unter Fachkräften im Gesundheits‐ und Körperpflegebereich zu reduzieren. Die Ergebnisse unterstreichen die Notwendigkeit einer weiteren Verbesserung der Intervention, rechtfertigen jedoch auch deren Umsetzung in Berufsbildungslehrplänen. Eine breitere Anwendung dieser Schulung könnte letztlich dazu beitragen, das weit verbreitete Stigma im Zusammenhang mit Hauterkrankungen zu bekämpfen und das Wohlbefinden, die soziale Teilhabe und die Lebensqualität der Betroffenen zu verbessern.

## DANKSAGUNG

Die Autoren möchten allen Teilnehmern und Patienten für ihre Zeit und Mühe sowie der Patientenorganisation Psoriasis‐Netz für ihre Unterstützung danken. Die Autoren danken außerdem dem Wissenschaftskommunikationsteam des Instituts, insbesondere Paula Willer, für die redaktionelle Bearbeitung.

Open access Veröffentlichung ermöglicht und organisiert durch Projekt DEAL.

## FINANZIERUNG

Diese Studie wurde von Eucerin, Beiersdorf AG, finanziert. Der Förderer hatte keinen Einfluss auf das Studiendesign, die Datenerhebung, die Datenanalyse, die Interpretation der Daten, die Erstellung des Berichts oder die Entscheidung, den Artikel zur Veröffentlichung einzureichen.

## INTERESSENKONFLIKT

M.A. war als Berater und/oder bezahlter Referent tätig und/oder hat Forschungsstipendien, Honorare und/oder Reisekostenerstattungen erhalten und/oder an von Unternehmen gesponserten klinischen Studien teilgenommen, darunter AbbVie, Almirall, Amgen, Beiersdorf AG, Biogen, BMS, Boehringer Ingelheim, Celgene, Centocor, Eli Lilly, Galderma, Janssen‐Cilag, LEO Pharma, medac, MSD, Novartis, Pfizer und Sandoz. D.R. ist derzeit bei der Beiersdorf AG beschäftigt. J.T., C.B.v.S., M.G. und R.S. haben keine Interessenkonflikte anzugeben.

## Supporting information



Supplementary information
