# Supplementary material for: Wirksamkeit einer Intervention zur Verringerung der Stigmatisierung von Menschen mit Hauterkrankungen unter Fachkräften im Gesundheits‐ und Körperpflegebereich – eine randomisierte kontrollierte Studie
Source: J Dtsch Dermatol Ges. 2026 Aug 4;24(8):1077–86. [Article in German] doi: 10.1111/ddg.16017_g (PMC13435003; doi:10.1111/ddg.16017_g)
Supplement: Supplementary file 1 [file DDG-24-1077-s001.docx]

**Wirksamkeit einer Intervention zur Verringerung der Stigmatisierung von Menschen mit Hauterkrankungen unter Fachkräften im Gesundheits- und Körperpflegebereich – eine randomisierte kontrollierte Studie - Zusatzmaterial**

**S1. Sekundäre Analysen**

*Verblindung hinsichtlich des Studienziels und der Gruppenzugehörigkeit.* Bei der Nachuntersuchung wurden die Teilnehmenden gebeten, zu beschreiben, was ihrer Meinung nach Ziel der Studie war, und anzugeben, ob sie glaubten, zur Interventionsgruppe oder zur Kontrollgruppe zu gehören, oder ob sie nicht wussten, zu welcher Gruppe sie gehörten.

*Kontrolle von Störfaktoren*. Um frühere Erfahrungen mit Hauterkrankungen zu kontrollieren, wurden die vier ANOVAs mit Messwiederholung zu den Auswirkungen von Gruppe und Zeit auf die primären Endpunkte mit den folgenden Kovariaten wiederholt: Betroffenheit von einer Hauterkrankung (ja/nein), Kenntnis einer anderen Person mit einer Hauterkrankung (ja/nein; basierend auf den Punkten 1–4 der Skala für gemeldetes und beabsichtigtes Verhalten ^1^) und Kontaktfrequenz zu Menschen mit einer Hauterkrankung (1=„nie“ bis 5=„täglich“).

*Feuermal*. Da der Interventionsinhalt so konzipiert war, dass er für alle Hauterkrankungen allgemein gültig ist, wurden die Fragebögen zur Bewertung der Zustimmung zu Stereotypen, krankheitsbezogener Fehlannahmen und des Wunsches nach sozialer Distanz ein zweites Mal mit Schwerpunkt auf Feuermalen durchgeführt, um zu testen, ob sich mögliche Veränderungen in stigmatisierenden Einstellungen auf andere Hauterkrankungen verallgemeinern lassen.

*Endpunkt Emotionale Reaktionen*. Eine Subskala der Desire for Social Distance Scale ^2^ bewertete emotionale Reaktionen auf drei Bilder von Personen mit Psoriasis und zwei Bilder von Personen mit Feuermalen. Die Bilder wurden mit schwarzem Hintergrund standardisiert, und die gezeigten Patienten hatten der Verwendung ihrer Bilder für Forschungszwecke zugestimmt. Die Teilnehmenden wurden gebeten, das Ausmaß, in dem sie jede von sechs Emotionen (Schuld, Mitgefühl, Verachtung, Neugier, Ekel, Mitleid) empfanden, auf einer Skala von 1=„zutreffend” bis 5=„nicht zutreffend” in Bezug auf die Bilder von Psoriasis und Feuermalen zu bewerten.

**S2. Subgruppenanalysen**

Tabelle S1 zeigt die Mittelwerte und Standardabweichungen der primären Endpunkte zu t0, t1 und t2 nach Beruf.

*Zustimmung zu negativen Stereotypen*. Es wurde ein signifikanter Haupteffekt der Zeit auf die Zustimmung zu negativen Stereotypen festgestellt, *F*(2,204)=10,51, *p*<0,001, η_p_^2^= 0,09. Es wurde kein Haupteffekt der Gruppe und keine signifikanten Interaktionseffekte festgestellt. Post-hoc-Tests zeigten, dass in der Interventionsgruppe die Zustimmung zu Stereotypen von t0 zu t1 (Differenz_t0-t1_: 0,538, *p*<0,001, 95 % KI 0,27-0,81) und t2 (Differenz_t0-t2_: 0,494, *p*<0,001, 95 % KI 0,18–0,81) signifikant abnahm, und zwischen t1 und t2 stabil blieb (Differenz_t1-t2_: -0,043 *p*=1,00, 95 % KI -0,33–0,25), allerdings nur bei den Physiotherapeuten. Bei den anderen Berufsgruppen gab es keine signifikanten Unterschiede zwischen den drei Zeitpunkten.

*Krankheitsbezogene Fehlannahmen*. Es wurden ein signifikanter Haupteffekt von Zeit, *F*(2,202)=25,13, *p*<0,001, η_p_^2^= 0,20, sowie signifikante Interaktionen zwischen Zeit und Gruppe, *F*(2,202)=15,11, *p*<0,001, η_p_^2^= 0,13 und zwischen Zeit und Beruf, *F*(4,202)=3,93, *p*=0,004, η_p_^2^= 0,07, beobachtet. Die Dreifach-Interaktion zwischen Zeit, Gruppe und Beruf war nicht signifikant. Post-hoc-Tests zeigten, dass in der Interventionsgruppe die krankheitsbezogenen Fehlannahmen von t0 zu t1 in allen Berufsgruppen signifikant abnahmen (Beauty: Differenz_t0-t1_: 0,687, *p*<0,001, 95 % KI 0,43-0,95); Physiotherapeuten: Differenz_t0-t1_: 0,426, *p*<0,001, 95 % KI 0,26-0,60); Pflegekräfte: Differenz_t0-t1_: 0,229, *p*=0,007, 95 % KI -0,05-0,41). Die Werte waren sowohl bei der Beauty-Gruppe als auch bei den Physiotherapeuten zu t2 im Vergleich zu t0 immer noch signifikant reduziert (Beauty: Differenz_t0-t2_: 0,267, *p*=0,018, 95 % KI 0,04–0,50; Physiotherapeuten: Differenz_t0-t2_: 0,380, *p*<0,001, 95 % KI 0,23-0,53), jedoch nicht bei den Pflegekräften (Differenz_t0-t2_: 0,035, *p*=1,00, 95 % KI -0,12-0,19). Die Beauty-Gruppe zeigte außerdem einen signifikanten Anstieg der krankheitsbezogenen Fehlannahmen von t1 zu t2 (Differenz_t1-t2_: -0,420, *p*=0,001, 95 % KI -0,70 bis (-0,14)), während dies bei den anderen Berufsgruppen nicht der Fall war (Physiotherapeuten: Differenz_t1-t2_: -0,046, *p*=1,00, 95 % KI -0,23-0,14; Pflegekräfte: Differenz_t1-t2_: -0,194, *p*=0,051, 95 % KI -0,39-0,00). In der Kontrollgruppe gab es zwischen den drei Zeitpunkten keine signifikanten Unterschiede.

*Wunsch nach sozialer Distanz*. Beim Wunsch nach sozialer Distanz gegenüber Menschen mit Psoriasis zeigte sich ein signifikanter Haupteffekt von Zeit, *Greenhouse-Geisser* *F*(1,77,180,557)=6,45, *p*=0,003, η_p_^2^=0,06, und ein Interaktionseffekt von Zeit x Beruf, *Greenhouse-Geisser* *F*(3,54, 180,557)=2,91, *p*=0,028, η_p_^2^=0,05. Post-hoc-Tests zeigten, dass in der Kontrollgruppe der Wunsch nach sozialer Distanz von t0 zu t1 (Differenz_t0-t1_: 0,233, *p*=0,006, 95 % KI 0,05-0,41) unter den Pflegekräften signifikant abnahm. Die Werte stiegen jedoch von t1 zu t2 signifikant an (Differenz_t1-t2_: -0,241, *p*=0,046, 95 % KI -0,48-(-0,01)) und unterschieden sich nicht mehr signifikant von t0 (Differenz_t0-t2_: -0,008, *p*=1,00, 95 % KI -0,25-0,24). Darüber hinaus nahm das Verlangen nach sozialer Distanz in der Physiotherapeutengruppe von t0 zu t1 signifikant ab (Differenz_t0-t1_: 0,154, *p*=0,048, 95 % KI 0,01–0,31) und blieb zwischen t1 und t2 stabil (Differenz_t1-t2_: -0,003, *p*=1,00, 95 % KI -0,21–0,20). In der Interventionsgruppe nahm das Verlangen nach sozialer Distanz in der Physiotherapeutengruppe von t0 zu t1 ebenfalls signifikant ab (Differenz_t0-t1_: 0,276, *p*<0,001, 95 % KI 0,11-0,44) und blieb zwischen t1 und t2 stabil (Differenz_t1-t2_: -0,09, *p*=0,987, 95 % KI -0,30-0,13).

*Berichtetes und beabsichtigtes Verhalten*. Es wurden keine signifikanten Haupt- oder Interaktionseffekte auf die Verhaltensabsichten festgestellt.

**Tabelle S1.** Deskriptive Statistik (Mittelwert, SD) der primären Endpunkte vor dem Seminar (t0), nach dem Seminar (t1) und bei der Nachuntersuchung (t2) sowie Veränderungen im Zeitverlauf nach Gruppe und Beruf.

| **Variable** | **Intervention (n=55)** | | | **Kontrolle (n=53)** | | |
| --- | --- | --- | --- | --- | --- | --- |
|  | **Mittelwert (SD)** | **Vergleich** | **Differenz** | **Mittelwert (SD)** | **Vergleich** | **Differenz** |
| **Beauty** | | |  |  |  |  |
| Zustimmung zu negativen Stereotypen | | |  |  |  |  |
| t0 | 2,82 (0,90) | t1-t0 | *p=*0,114 | 2,76 (0,78) | t1-t0 | *p=*1,000 |
| t1 | 2,46 (0,77) | t2-t0 | *p=*1,000 | 2,75 (1,02) | t2-t0 | *p=*0,708 |
| t2 | 2,65 (0,57) | t2-t1 | *p=*0,943 | 2,50 (0,79) | t2-t1 | *p=*0,649 |
| Wunsch nach sozialer Distanz | |  |  |  |  |  |
| t0 | 1,75 (0,70) | t1-t0 | *p=*0,153 | 1,72 (0,65) | t1-t0 | *p=*0,476 |
| t1 | 1,55 (0,60) | t2-t0 | *p=*1,000 | 1,88 (0,88) | t2-t0 | *p=*0,063 |
| t2 | 1,79 (0,44) | t2-t1 | *p=*0,248 | 2,09 (1,27) | t2-t1 | *p=*0,525 |
| Krankheitsbezogene Fehlannahmen | | |  |  |  |  |
| t0 | 2,48 (0,72) | t1-t0 | *p <*0,001 | 2,39 (0,52) | t1-t0 | *p=*0,633 |
| t1 | 1,79 (0,34) | t2-t0 | *p=*0,018 | 2,24 (0,53) | t2-t0 | *p=*0,259 |
| t2 | 2,21 (0,82) | t2-t1 | *p=*0,001 | 2,21 (0,49) | t2-t1 | *p=*1,000 |
| Verhaltensabsichten | |  |  |  |  |  |
| t0 | 17,00 (5,21) | t1-t0 | *p=*0,829 | 18,14 (2,55) | t1-t0 | *p=*0,986 |
| t1 | 16,20 (6,56) | t2-t0 | *p=*0,209 | 17,25 (3,06) | t2-t0 | *p=*0,827 |
| t2 | 15,80 (4,87) | t2-t1 | *p=*1,000 | 16,13 (4,19) | t2-t1 | *p=*1,000 |
| **Pflegekräfte** | | | | | | |
| Zustimmung zu negativen Stereotypen | | | | | | |
| t0 | 2,67 (0,63) | t1-t0 | *p=*0,079 | 2,77 (0,50) | t1-t0 | *p=*0,046 |
| t1 | 2,41 (0,70) | t2-t0 | *p=*0,750 | 2,47 (0,53) | t2-t0 | *p=*1,000 |
| t2 | 2,52 (0,71) | t2-t1 | *p=*1,000 | 2,66 (0,54) | t2-t1 | *p=*0,467 |
| Wunsch nach sozialer Distanz | | | | | | |
| t0 | 1,58 (0,53) | t1-t0 | *p=*0,657 | 1,77 (0,52) | t1-t0 | *p=*0,006 |
| t1 | 1,50 (0,49) | t2-t0 | *p=*1,000 | 1,53 (0,40) | t2-t0 | *p=*1,000 |
| t2 | 1,52 (0,57) | t2-t1 | *p=*1,000 | 1,77 (0,44) | t2-t1 | *p=*0,046 |
| Krankheitsbezogene Fehlannahmen | | | | | | |
| t0 | 1,95 (0,36) | t1-t0 | *p=*0,007 | 1,88 (0,34) | t1-t0 | *p=*1,000 |
| t1 | 1,72 (0,29) | t2-t0 | *p=*1,000 | 1,87 (0,39) | t2-t0 | *p=*1,000 |
| t2 | 1,91 (0,43) | t2-t1 | *p=*0,051 | 1,91 (0,31) | t2-t1 | *p=*1,000 |
| Verhaltensabsichten | | | | | | |
| t0 | 18,95 (1,73) | t1-t0 | *p=*1,000 | 18,21 (1,93) | t1-t0 | *p=*1,000 |
| t1 | 18,77 (2,39) | t2-t0 | *p=*1,000 | 18,53 (1,90) | t2-t0 | *p=*1,000 |
| t2 | 18,48 (1,63) | t2-t1 | *p=*1,000 | 18,42 (2,52) | t2-t1 | *p=*1,000 |
| **Physiotherapeutinnen** | | | | | | |
| Zustimmung zu negativen Stereotypen | | | | | | |
| t0 | 2,75 (0,51) | t1-t0 | *p <*0,001 | 2,77 (0,65) | t1-t0 | *p=*1,000 |
| t1 | 2,22 (0,57) | t2-t0 | *p <*0,001 | 2,67 (0,64) | t2-t0 | *p=*1,000 |
| t2 | 2,26 (0,57) | t2-t1 | *p=*1,000 | 2,66 (0,68) | t2-t1 | *p=*1,000 |
| Wunsch nach sozialer Distanz | | | | | | |
| t0 | 1,55 (0,35) | t1-t0 | *p <*0,001 | 1,75 (0,62) | t1-t0 | *p=*0,048 |
| t1 | 1,27 (0,26) | t2-t0 | *p=*0,126 | 1,59 (0,53) | t2-t0 | *p=*0,252 |
| t2 | 1,36 (0,32) | t2-t1 | *p=*0,987 | 1,60 (0,56) | t2-t1 | *p=*1,000 |
| Krankheitsbezogene Fehlannahmen | | | | | | |
| t0 | 2,09 (0,33) | t1-t0 | *p <*0,001 | 2,12 (0,43) | t1-t0 | *p=*1,000 |
| t1 | 1,67 (0,19) | t2-t0 | *p <*0,001 | 2,11 (0,42) | t2-t0 | *p=*1,000 |
| t2 | 1,71 (0,26) | t2-t1 | *p=*1,000 | 2,10 (0,41) | t2-t1 | *p=*1,000 |
| Verhaltensabsichten | | | | | | |
| t0 | 18,61 (1,47) | t1-t0 | *p=*1,000 | 17,77 (2,58) | t1-t0 | *p=*0,933 |
| t1 | 19,04 (1,58) | t2-t0 | *p=*1,000 | 17,31 (3,52) | t2-t0 | *p=*1,000 |
| t2 | 19,00 (1,48) | t2-t1 | *p=*1,000 | 18,08 (2,34) | t2-t1 | *p=*0,383 |

**S3. Vorherige Erfahrungen mit Hauterkrankungen**

*Eigene Betroffenheit von einer Hauterkrankung.* Die ANCOVAs mit Messwiederholung zur Zustimmung zu negativen Stereotypen, dem Wunsch nach sozialer Distanz und krankheitsbezogenen Fehlannahmen in Bezug auf Psoriasis und Feuermale sowie zu Verhaltensabsichten wiesen alle keine signifikanten Effekte durch eigene Betroffenheit auf (alle *p*>0,06). Die Effekte von Zeit und Gruppe auf die verschiedenen Endpunkte waren zwischen den Teilnehmenden, die angaben, selbst eine Hauterkrankung zu haben (n=18), und denen, die angaben, keine zu haben, vergleichbar.

*Bekanntschaft mit einer Person mit einer Hauterkrankung*. Die ANCOVAs mit Messwiederholung zur Zustimmung zu negativen Stereotypen, Wunsch nach sozialer Distanz und Verhaltensabsichten wiesen keine signifikanten Effekte der Bekanntschaft mit Personen mit einer Hauterkrankung auf (alle *p*>0,08). Wichtig ist, dass die Bekanntschaft mit einer anderen Person mit einer Hauterkrankung die Ausprägung krankheitsbezogener Fehlannahmen in Bezug auf Psoriasis (*p*=0,002), jedoch nicht in Bezug auf Feuermale (*p*=0,513) bedingte. Der Effekt von Gruppe auf krankheitsbezogene Fehlannahmen in Bezug auf Psoriasis blieb auch nach Bereinigung um die Bekanntschaft mit einer anderen Person mit einer Hauterkrankung signifikant, *F*(1,104)=5,535, *p*=0,021.

*Kontaktfrequenz*. Eine höhere Kontaktfrequenz bedingte signifikant einen geringeren Wunsch nach sozialer Distanz gegenüber Personen mit Psoriasis (*p*=0,024) und Feuermalen (*p*=0,029), weniger krankheitsbezogene Fehlannahmen über Psoriasis (*p*<0,001) und positivere Verhaltensabsichten gegenüber Personen mit einer Hauterkrankung (*p*<0,001). Die Effekte von Gruppe auf den Wunsch nach sozialer Distanz gegenüber Menschen mit Psoriasis, *F*(1,103)=4,121, *p*=0,045, und auf krankheitsbezogene Fehlannahmen über Psoriasis, *F*(1,102)=4,709, *p*=0,032, blieben beide nach Kontrolle der Kontaktfrequenz signifikant. Die Effekte von Gruppe auf den Wunsch nach sozialer Distanz gegenüber Personen mit Feuermal, F(1,102)=1,282, *p*=0,260, und auf Verhaltensabsichten, *F*(1,102)=0,996, *p*=0,321, blieben nach Bereinigung um die Kontaktfrequenz nicht signifikant. Die rmANCOVAs zu Zustimmung zu negativen Stereotypen (Psoriasis, Feuermal) und zu krankheitsbezogenen Fehlannahmen über Feuermale wiesen keine signifikanten Effekte der Kontaktfrequenz auf (alle *p*>0,60).

**S4. Feuermal Analysen**

*Zustimmung zu negativen Stereotypen*. Es wurden keine Haupteffekte von Zeit, *Huynh-Feldt* *F*(1,84,195,09)=0,50, *p*=0,592, oder Gruppe auf Zustimmung zu negativen Stereotypen, *F*(1,106)=3,37, *p*=0,069, noch ein Interaktionseffekt, *Huynh-Feldt* *F*(1,84,195,09)=1,21, *p*=0,297, festgestellt. Darüber hinaus zeigte die Subgruppenanalyse keine Interaktion mit Beruf, was darauf hindeutet, dass die Ergebnisse über die drei Berufe hinweg vergleichbar waren.

*Krankheitsbezogene Fehlannahmen*. Die Haupteffekte von Zeit, *F*(2,210)=2,652, *p*=0,073, und Gruppe, *F*(1,105)=0,27, *p*=0,606, auf krankheitsbezogene Fehlannahmen waren nicht signifikant. Es zeigte sich jedoch ein signifikanter Interaktionseffekt, *F*(2,210)=4,845, *p*=0,009, η_p_^2^= 0,04. Post-hoc-Tests zeigten, dass in der Interventionsgruppe die krankheitsbezogenen Fehlannahmen von t0 zu t2 signifikant abnahmen (Differenz_t0-t2_: 0,241, *p*=0,003, 95 % KI 0,07-0,41). Der Rückgang von t0 zu t1 war nicht signifikant (Differenz_t0-t1_: 0,199, *p*=0,052, 95 % KI -0,001-0,40). In der Kontrollgruppe gab es keine signifikanten Unterschiede zwischen den drei Zeitpunkten. Die Subgruppenanalyse ergab einen signifikanten Interaktionseffekt zwischen Zeit und Gruppe, *F*(2,202)=4,07, *p*=0,019, η_p_^2^=0,04. Es wurden keine weiteren Haupt- oder Wechselwirkungseffekte festgestellt. Post-hoc-Tests zeigten einen signifikanten Rückgang der krankheitsbezogenen Fehlannahmen bei den Pflegekräften in der Interventionsgruppe zwischen t0 und t2 (Differenz_t0-t2_: 0,298, *p*=0,029, 95 % KI 0,02-0,57). Bei den anderen Berufsgruppen und in der Kontrollgruppe wurden keine signifikanten Veränderungen beobachtet.

*Wunsch nach sozialer Distanz*. Es wurden keine Haupteffekte von Zeit, *Huynh-Feldt* *F*(1,68,176,50)=0,035, *p*=0,946, oder Gruppe, *F*(1,105)=1,996, *p*=0,161, auf den Wunsch nach sozialer Distanz noch ein Interaktionseffekt, *Huynh-Feldt* *F*(1,68,176,50)=1,583, *p*=0,211, festgestellt. Die Subgruppenanalyse ergab keine Interaktionen mit Beruf, was darauf hindeutet, dass die Ergebnisse über die drei Berufe hinweg vergleichbar waren.

**S5. Analysen zu emotionalen Reaktionen**

*Emotionale Reaktionen auf Bilder von Psoriasis.* Es wurde ein signifikanter Haupteffekt von Zeit, *Greenhouse-Geisser* *F*(1,52,161,51)=8,65, *p*<0,001, auf Mitgefühl festgestellt, während der Haupteffekt von Gruppe, *F*(1,106)=0,02, *p*=0,898, und der Interaktionseffekt nicht signifikant waren, *Greenhouse-Geisser* *F(*2,210)=1,15, *p*=0,309. Post-hoc-Tests zeigten, dass in der Interventionsgruppe Mitgefühl von t0 (Differenz_t0-t2_: 0,309, *p*=0,005, 95 % KI 0,08-0,54) und t1 (Differenz_t1-t2_: 0,297, *p*=0,012, 95 % KI 0,05–0,54) zu t2 deutlich abgenommen hatte, während zwischen t0 und t1 kein Unterschied bestand (Differenz_t0-t1_: 0,012, *p*=1,00, 95 % KI -0,13–0,15). In der Kontrollgruppe gab es keine signifikanten Unterschiede zwischen den drei Zeitpunkten. Ebenso wurde ein signifikanter Haupteffekt von Zeit, *Huynh-Feldt* *F*(1,81,191,99)=4,98, *p*=0,010, für Mitleid festgestellt, während der Haupteffekt von Gruppe, *F*(1,106)=0,03, *p*=0,863, und der Interaktionseffekt nicht signifikant waren, *Huynh-Feldt* *F*(1,81,191,99)=0,27, *p*=0,745. Post-hoc-Tests zeigten, dass in der Kontrollgruppe das Mitleid von t0 zu t2 signifikant abnahm (Differenz_t0-t2_: 0,289, *p*=0,037, 95 % KI 0,01-0,57), während es keinen Unterschied zwischen t0 und t1 (Differenz_t0-t1_: 0,201, *p*=0,096, 95 % KI -0,02-0,43) oder zwischen t1 und t2 (Differenz_t1-t2_: 0,088, *p*=1,00, 95 % KI -0,23-0,40) gab. Es gab keine signifikanten Unterschiede zwischen den drei Zeitpunkten in der Interventionsgruppe. Ein signifikanter Haupteffekt von Zeit, *Huynh-Feldt* *F(*1,86,196,76)=4,32, *p*=0,017, auf Schuldzuweisung wurde ebenfalls festgestellt, während der Haupteffekt von Gruppe, *F*(1,106)=0,68, *p*=0,413, und der Interaktionseffekt nicht waren signifikant, *Huynh-Feldt* *F(*1,86,196,76)=1,02, *p*=0,359. Post-hoc-Tests zeigten, dass in der Interventionsgruppe die Schuldzuweisung von t0 zu t1 signifikant abnahm (Differenz_t0-t1_: 0,273, *p*=0,014, 95 % KI 0,04-0,50) und von t1 zu t2 wieder signifikant anstieg (Differenz_t1-t2_: -0,333, *p*=0,026, 95 % KI -0,63-(-0,03)), während zwischen t0 und t2 kein Unterschied bestand (Differenz_t0-t2_: -0,061, *p*=1,00, 95 % KI -0,36-0,24). In der Kontrollgruppe gab es keine signifikanten Unterschiede zwischen den drei Zeitpunkten. Darüber hinaus wurden signifikante Haupteffekte von Zeit, *Huynh-Feldt* *F*(1,94,205,88)=30,66, *p*<0,001, und Gruppe, *F*(1,106)=5,09, *p*=0,026, auf Ekel festgestellt, während der Interaktionseffekt nicht signifikant war, *Huynh-Feldt* *F(*1,94,205,88)=2,02, *p=*0,136. Post-hoc-Tests zeigten, dass in der Kontrollgruppe Ekel von t0 zu t1 signifikant abnahm (Differenz_t0-t1_: 0,541, *p=*0,01, 95 % KI 0,19-0,90) und von t1 zu t2 wieder signifikant zunahm (Differenz_t1-t2_: -0,321, *p=*0,023, 95 % KI -0,61-(-0,03)), während zwischen t0 und t2 kein Unterschied bestand (Differenz_t0-t2_: 0,220, *p=*0,313, 95 % KI -0,11-0,55). In der Interventionsgruppe nahm Ekel von t0 zu t1 signifikant ab (Differenz_t0-t1_: 0,915, *p* <0,001, 95 % KI 0,57–1,26) und stieg von t1 zu t2 wieder signifikant an [Differenz_t1-t2_: -0,539, *p* <0,001, 95 % KI -0,82 (-0,26)]. Dennoch war das Ekelniveau zu t2 immer noch signifikant niedriger als zu t0 (Differenz_t0-t2_: 0,376, *p=*0,016, 95 % KI 0,06-0,70). Die Haupteffekte von Zeit, *F(*2,212)=0,76, *p=*0,468, und Gruppe, *F(*1,106)=0,98, *p=*0,324, auf Neugier waren nicht signifikant, aber es zeigte sich ein signifikanter Interaktionseffekt, *F(*2,212)=5,19, *p=*0,006. Post-hoc-Tests zeigten, dass in der Kontrollgruppe die Neugierde von t0 zu t2 signifikant abnahm (Differenz_t0-t2_: 0,409, *p=*0,020, 95 % KI 0,05-0,77), während es keinen Unterschied zwischen t0 und t1 (Differenz_t0-t1_: 0,270, *p*=0,105, 95 % KI -0,04-0,58) oder zwischen t1 und t2 (Differenz_t1-t2_: 0,055, *p*=1,00, 95 % KI -0,30-0,41) gab. Es gab keine signifikanten Unterschiede zwischen den drei Zeitpunkten in der Interventionsgruppe. Schließlich wurden keine Haupt- oder Interaktionseffekte auf Verachtung festgestellt.

*Emotionale Reaktionen auf Bilder von Feuermalen*. Es wurde ein signifikanter Haupteffekt von Zeit, *F*(2,210)=4,11, *p*=0,018, auf Mitgefühl festgestellt, während der Haupteffekt von Gruppe, *F*(1,105)=0,20 *p*=0,658, und der Interaktionseffekt nicht signifikant waren, *F*(2,210)=1,08, *p*=0,342. Post-hoc-Tests ergaben keine signifikanten Unterschiede zwischen den drei Zeitpunkten für beide Gruppen. Ein signifikanter Haupteffekt von Zeit, *Huynh-Feldt* *F*(1,89,198,78)=5,66, *p*=0,005, auf Ekel wurde ebenfalls festgestellt, während der Haupteffekt von Gruppe, *F*(1,105)=3,58, *p*=0,061, und der Interaktionseffekt nicht signifikant waren, *Huynh-Feldt* *F*(1,89,198,78)=1,18, *p*=0,311. Post-hoc-Tests zeigten, dass in der Interventionsgruppe das Ekelempfinden von t1 zu t2 signifikant zunahm [Differenz_t1-t2_: -0,333, *p*=0,007, 95 % KI -0,59-(-0,07)]. Die Ekelgefühle zu t1 (Differenz_t0-t1_: 0,185, *p*=0,108, 95 % KI -0,03-0,40) und t2 (Differenz_t0-t2_: -0,148, *p*=0,577, 95 % KI -0,42-0,13) unterschieden sich nicht signifikant von t0. In der Kontrollgruppe gab es keine signifikanten Unterschiede zwischen den drei Zeitpunkten. Es wurden keine Haupt- oder Interaktionseffekte in Bezug auf Schuld, Verachtung, Neugier oder Mitleid festgestellt.

**Referenzen**

1	Evans-Lacko S, Rose D, Little K, Flach C, Rhydderch D, Henderson C *et al.* Development and psychometric properties of the reported and intended behaviour scale (RIBS): a stigma-related behaviour measure. *Epidemiol Psychiatr Sci* 2011;20:263–71.

2	Pearl RL, Wan MT, Takeshita J, Gelfand JM. Stigmatizing attitudes toward persons with psoriasis among laypersons and medical students. *J Am Acad Dermatol* 2019;80:1556–63.
